# Oncologic outcomes of laparoscopic gastrectomy: a single-center safety and feasibility study

**DOI:** 10.1007/s00464-012-2696-3

**Published:** 2013-03-07

**Authors:** Nobuhisa Matsuhashi, Shinji Osada, Kazuya Yamaguchi, Shiro Saito, Naoki Okumura, Yoshihiro Tanaka, Kenichi Nonaka, Takao Takahashi, Kazuhiro Yoshida

**Affiliations:** Surgical Oncology, Gifu University School of Medicine, 501-1194 1-1 Yanagido, Gifu, Japan

**Keywords:** Laparoscopic surgery, Laparoscopic gastrectomy, Lymph node dissection

## Abstract

**Background:**

Indications for laparoscopic gastrectomy (LG) for early stomach cancer have spread worldwide and evaluation of short-term outcomes has been favorable. The present study aimed to evaluate both technical feasibility and safety of LG and short-and long-term outcomes after LG.

**Methods:**

The study group comprised 231 patients who underwent LG during the period from August 2001 through December 2011 at Gifu University School of Medicine.

**Results:**

Concomitant resection of other organs was performed in 16 (6.9 %) of the 231 patients, and conversion to open surgery was performed in 5 (2.2 %) patients. The final clinical stage of the patients, according to the Union for International Cancer Control classification, was stage IA in 183 (79.0 %), stage IB in 26 (11.3 %), stage IIA in 9 (2.6 %), stage IIB in 6 (2.6 %), stage IIIA in 5 (2.2 %), and stage IIIB in 2 (0.9 %) patients. Average values of total blood loss and operation time were 133.7 ± 129.0 ml and 328.1 ± 70.1 min, respectively. Postoperative complications were detected in 29 patients (12.6 %), and one patient died. According to the Clavien–Dindo classification of surgical complications, the rate of severe complications of grade ≥3a was 6.1 % and that of grade ≥3b was 1.3 %. There were no significant differences in complications in relation to clinicopathological or operative procedures. Cancer recurrence was detected in 2 (0.9 %) patients. In the patient with peritoneal dissemination, tumor size and macroscopic type were critical. Five-year overall survival rates were 99.3 % for stage IA, 95.2 % for stage IB, and 50.0 % for stage IIB patients. One recurrence each was detected for stages IA and IIB cancers.

**Conclusion:**

The present study showed LG to have a safe postoperative course and to benefit oncologic outcomes.

Since the first report on laparoscopy-assisted distal gastrectomy by Kitano et al. [1], laparoscopic surgery, including that for gastrectomy or colectomy for early stage cancer, has developed worldwide during the last several years [2, 3]. Laparoscopic gastrectomy (LG) has become commonly accepted because it results in less wound pain, quicker recovery, and a shorter hospital stay [4]. Novel surgical procedures and instruments have also improved safety and assured reduced postoperative complications [5]. Indeed, according to recent clinical trials from 16 surgical departments of the Japanese Laparoscopic Surgery Study Group [2], morbidity and mortality rates after LG were 14.8 and 0 %, respectively. Recently, laparoscopic surgery has been selected as a first therapeutic procedure because of high-quality evidence for short-term outcomes, but long-term efficacy with respect to delayed complications and prognosis, including recurrence, has not yet been determined. A previous single-center experience with 601 patients who underwent LG is the only report to show both short- and long-term results [6]; it demonstrated rates of morbidity, mortality, and recurrence of 17.6 %, 0.3 %, and 2.5 %, respectively. In the present study, we investigated not only the technical feasibility and safety of LG but also short- and long-term outcomes after the procedure.

## Patients and clinical evaluations

### Patients

This study comprised 231 patients with gastric cancer (159 men, 72 women; mean age = 64.8 ± 11.0 years) who underwent LG in the surgical oncology department of Gifu University School of Medicine from August 2001 to December 2011. Mean body mass index (BMI) of the study patients was 22.1 ± 3.5 kg/m^2^. Patient clinicopathological features are given in Table 1.

**Table 1:** Demographic, operative, and tumor characteristics of 231 patients after laparoscopic radical gastrectomy

Patients	*n* = 231
Age (years)	64.8 ± 11.0
Gender (male/female)	159/72
Body mass index	22.1 ± 3.5
Operation	
Totally laparoscopic procedure	77.90 %
Assisted/not assisted	51/180
Conversions	5
Type of gastrectomy	
DG/PPG/PG/TG	186/8/26/11
D1/D1 + α/D1 + β/D2	2/52/151/26
Lymph node yields	29.2 ± 15.8
Resection of other organ	
Yes/no	15/216
Operation time (min)	328.1 ± 70.1
Postoperative days	15.2 ± 8.4
Bleeding (ml)	133.7 ± 129.0
Tumor histology	
pap/tub1/tub2/por1/por2/sig/muc/end	4/63/64/28/30/40/1/1
Tumor size (cm)	3.4 ± 2.1
Stage	
Understaging	19/231 (8.2 %)
IA/IB/IIA/IIB/IIIA/IIIB/IIIC	183/27/8/6/5/2/0

Pathological study showed all tumors to be malignant, and preoperative imaging procedures indicated that there was no spread over the muscularis propria (MP) with N0 lymph node metastases. Our policy for such cancer progression has usually been to perform D1+ lymph node dissection. The operative method was converted to open surgery if serosal invasion or extensive lymphadenopathy was detected at laparoscopy.

Written informed consent was obtained from all patients enrolled in the study. The study protocol conformed to the ethical guidelines of the 1975 Declaration of Helsinki and the guidelines of the regional ethical committees of Zurich and Basel, Switzerland.

### Operative indications and procedures

According to the location of the tumor, the laparoscopic procedure was either distal gastrectomy (LDG), proximal gastrectomy (LPG), pylorus-preserving gastrectomy (LPPG), or total gastrectomy (LTG). LPG was used for lesions in the upper third of the stomach with no serosal invasion or lymph node involvement. LPPG was selected for tumors <4 cm in diameter located in the stomach body at least 4 cm from the pylorus and with limited mucosal invasion or submucosal invasion of <2 cm. LTG was performed for locally advanced proximal lesions or multiple lesions for which the distal stomach could not be preserved.

As described by Kitano et al. [3], LG consists of the following procedures: (1) laparoscopic dissection of the lesser and greater omentum and ligation and division of the main vessels to mobilize the stomach under pneumoperitoneum, (2) laparoscopic D1 + α, D1 + β, or D2 lymph node dissection based on the Guidelines of the Japanese Gastric Cancer Association, and (3) resection of the stomach according to the location of the tumor as above, followed by reconstruction by the Billroth I, esophagogastrostomy, or Roux-en-Y method. The technical procedure of LG used in our institution was accepted by the Japan Society for Endoscopic Surgery (JSES) after reviewing a video presentation of the procedure.

All patients were monitored for recurrence and general condition postoperatively by blood examination, including tumor markers such as serum carcinoembryonic antigen, and by imaging such as computed tomography at least every 3 months for the first year and every 6 months after 2 years along with gastroscopy once a year. The median follow-up period was 44.4 ± 27.6 months (range = 3–118 months). Total number of patients that reached 5 years was 88 patients (38.1 %). According to the results in Japan [7–10], in which postoperative adjuvant therapy with the drug S-1 improved overall survival and relapse-free survival in patients with stages II or III gastric cancer, the present patients also underwent the same therapy.

### Statistical analysis

All data are presented as mean ± SD. The data were evaluated statistically using the Student *t* test, Wilcoxon signed-rank test, Kaplan–Meier method, log-rank test, and Pearson product-moment correlation coefficient to determine statistical significances. A value of *p* < 0.05 was regarded as indicating statistical significance.

### Clavien–Dindo classification

We evaluated feasibility and safety of the procedure with the Clavien–Dindo classification, which categorizes surgical complications from grades 1 to 5 based on the invasiveness of the treatment required. Grade 1 requires no treatment; grade 2 requires medical therapy; grade 3a requires surgical, endoscopic, or radiologic intervention but not general anesthesia; grade 3b requires general anesthesia; grade 4 represents life-threatening complications that require intensive care; and grade 5 represents death of the patient. In this study we retrospectively determined complications ranging from grades 2 to 5 from patient records during hospitalization and within 30 days after surgery. Grade 1, except for surgical site infection, was not evaluated to exclude the possibility of description bias in the patient records. Severe complications were defined as those graded as ≥3a. Mortality (grade 5) was defined as hospital death due to any cause after surgery [11].

## Results

Of the 231 patients, 186 (80.5 %) were selected for LDG, 8 (3.5 %) for PPG, 26 (11.3 %) for PG, and 11 (4.89 %) for TG. Concomitant resections of other organs were performed in 16 (6.9 %) patients and consisted of 13 cholecystectomies for gallstones, 1 appendectomy, 1 rectal resection for synchronous rectal cancer, and 1 herniorrhaphy. Conversion to open surgery was performed in 5 (2.2 %) patients because of advanced-stage disease in 2 patients, uncontrollable hemorrhage in 1, serious adhesions in 1, and positive margin in 1 patient. The final clinical stage of patients according to the Union for International Cancer Control (UICC) classification was stage IA in 186 (79.2 %), stage IB in 26 (11.3 %), stage IIA in 9 (2.6 %), stage IIB in 6 (2.6 %), stage IIIA in 5 (2.2 %), and stage IIIB in 2 (0.9 %) patients. The average values for total blood loss and operation time were 133.7 ± 129.0 ml and 328.1 ± 70.1 min, respectively.

Postoperative complications were detected in 29 (12.6 %) patients and included anastomotic stenosis in 2 (0.9 %) patients, anastomotic leakage in 3 (1.3 %), pancreatic injury in 8 (3.5 %), and wound infection in 3 (1.3 %) patients. One patient died from severe sepsis. Postoperative complications detected for each surgical procedure are shown in Table 2.

**Table 2:** Intraoperative and postoperative complications in 231 patients who underwent laparoscopic radical gastrectomy

Complications	Type of resection				
	DG (*n* = 186)	PPG (*n* = 8)	PG (*n* = 26)	TG (*n* = 11)	Total (*n* = 231)
Morbidity					
Intraoperative [*n* (%)]	5 (2.7)	0	0	0	5 (2.2)
Bleeding [*n* (%)]	3 (1.6)	0	0	0	3 (.1.3)
Organ injury [*n* (%)]	2 (1.1)	0	0	0	2 (0.9)
Postoperative [*n* (%)]	23 (12.4)	0 (0)	4 (15.4)	2 (18.2)	29 (12.6)
Anastomotic leakage [*n* (%)]	2 (1.1)	0	1 (3.8)	0	3 (1.3)
Duodenal stump leakage (*n*)	0	0	0	0	0
Anastomotic stricture [*n* (%)]	2 (1.1)	0	0	0	2 (0.9)
Anastomotic ulcer [*n* (%)]	1 (0.5)	0	0	0	1 (0.4)
Stasis (*n*)	0	0	0	0	0
Pancreatic injury [*n* (%)]	7 (3.8)	0	1 (3.8)	0	8 (3.5)
Bleeding (*n*)	0	0	0	0	0
Bowel obstruction [*n* (%)]	4 (2.2)	0	1 (3.8)	1 (9.1)	6 (2.6)
Abdominal abscess [*n* (%)]	3 (1.6)	0	0	0	3 (1.3)
Wound infection [*n* (%)]	2 (1.1)	0	1 (3.8)	0	3 (1.3)
Pulmonary infection [*n* (%)]	2 (1.1)	0	0	0	2 (0.9)
Urinary infection [*n* (%)]	0	0	0	1 (9.1)	1 (0.4)
Mortality					
Severe sepsis [*n* (%)]	1 (0.5)	0	0	0	1 (0.5)

Complications were classified as grade 2 in 13 patients, grade 3a in 11 patients, grade 3b in 2 patients, grade 4 in 0 patients, and grade 5 in 1 patient. The rate of severe complications of grade ≥3a was 6.1 % and that of grade ≥3b was 1.3 % (Table 3).

**Table 3:** Details of patients with Clavien–Dindo classification

Complications	Grade 1	Grade 2	Grade 3a	Grade 3b	Grade 4	Grade 5
Anastomotic leakage (*n*)		2	1			
Anastomotic stricture (*n*)		1	1			
Anastomotic ulcer (*n*)		1				
Pancreatic injury (*n*)		4	3	1		
Bowel obstruction (*n*)		1	4	1		
Abdominal abscess (*n*)		1	2			
Wound infection (*n*)	3					
Pulmonary infection (*n*)		2				
Urinary infection (*n*)		1				
Severe sepsis						1
Total	3	13	11	2	0	1

There were no significant differences in complications in relation to clinicopathological features or operative procedures. Local invasions of the stomach wall in the patients with recurrence were into the submucosal layer and serosal layer without histological lymph node metastases (N0) and were classified as stages IA and IIB. Recurrence was not associated with operative indication, surgical procedure, or postoperative complications.

Cancer recurrence was detected in 2 (0.9 %) patients: one in lymph node with liver metastasis after 34 months and one in peritoneal dissemination after 10 months (Table 4). In the patient with peritoneal dissemination, the tumor size was 12 cm and was type 4 macroscopically with esophageal invasion, despite a preoperative evaluation as type IIc. This patient was converted to open laparotomy to perform total gastrectomy with D2 lymph node dissection.

**Table 4:** Details of patients with distant and local recurrences after laparoscopic radical gastrectomy

Patients no.	Age (years)/gender	Types of LG	Intervals after LG (months)	Recurrence site	Preoperative diagnosis stage	Pathologic findings of primary disease							
						His	Size (mm)	Depth	ly	v	*N*	UIC stage	Outcomes
1	70/female	TG	10	P	IB	por2	120	T4a	ly1	v1	0	IIB	Dead
2	64/male	PG	34	H, L	IA	tub1	25	T1b	ly1	v2	0	IA	Alive

**Fig. 1:**
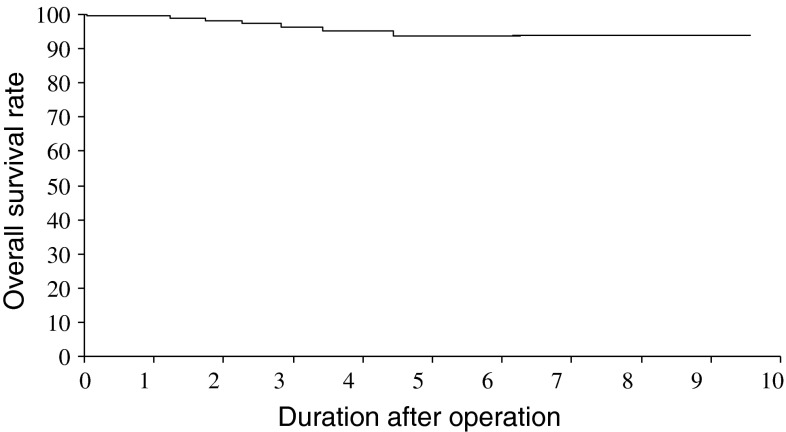
Kaplan–Meier overall patient survival curves. The present whole cases were detected

**Fig. 2:**
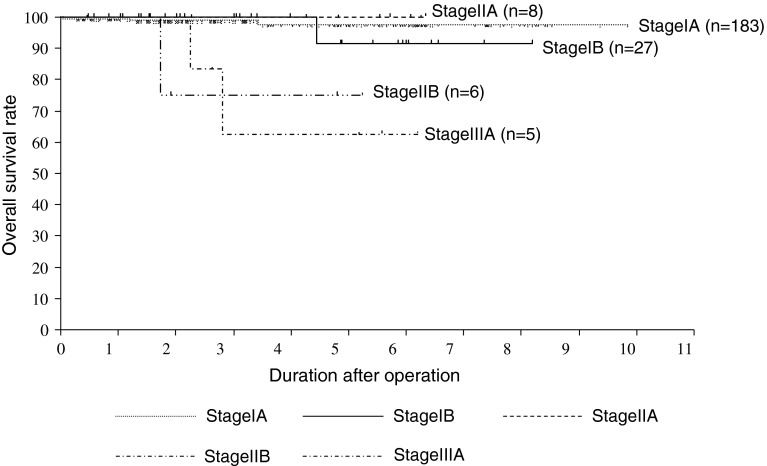
Kaplan–Meier overall patient survival curves. Based on the 7th edition of the Union for International Cancer Control classification, each stage curve is shown. Two patients with stage IIIB disease were omitted because of small sample size

Cumulative survival curves are shown in Fig. 1. The 5-year overall survival rates were 99.3 % for stage IA, 95.2 % for stage IB, and 50.0 % for stage IIB patients (Fig. 2). Recurrence was detected in one stage IA and one stage IIB patient. Five patients died due to other reasons.

## Discussion

In this decade, developments in surgical procedure and instruments have expanded the operative indication of LG from limited early stage to relatively advanced cancer. As a first step toward acceptance after comparison with open surgery, LG showed not only several advantages, such as less pain, earlier recovery of bowel movement, and shorter hospital stay [2, 3], but also fewer complications [4, 5]. Five (2.2 %) patients in the present study suffered intraoperative complications. A recent single-center report demonstrated an intraoperative complication rate of 11.1 % [6], indicating that the rate in the present study might be satisfactory. The most frequent postoperative complication was pancreatic injury, but its rate of occurrence was not different for each of the surgical procedures performed. In a previous study, pancreatic injury was reported to be higher with D2 rather than D1 lymph node dissection [12], and the occurrence of pancreatic fistula was much more frequent if D1 dissection with suprapancreatic lymph node site was added [13]. Although lymph node dissection in the present study was based on the Guidelines of the Japanese Gastric Cancer Association [13], our policy led to aggressive lymph node resection being commonly selected even if laparoscopic surgery was performed, and, in fact, 26 (11.3 %) of the 231 procedures were performed with D2 lymph node dissection. The rate of pancreatic injury was previously reported to be 1.0 % [2] or 11.1 % [6], and it was 3.8 % in the present study. One of the most critical factors for pancreatic injury might be a BMI of ≥25 % [14]. In addition, pancreatic injury is one of the most critical problems with LG, and it is suggested that it results from laparoscopic coagulating shears during lymph node dissection, especially when expanding the area.

Blood loss and operative times in the present study were both similar to those of past reports, which described ranges of 112–336 ml and 196–348 min, respectively [15–18]. Furthermore, the morbidity and mortality rates of the present study, 12.6 and 0.3 %, were also similar to those in previous studies [19, 20].

Many retrospective studies have not defined complications. Recently, Dindo et al. [11] proposed a new classification of surgical complications called the Clavien–Dindo classification. Many retrospective studies have also not mentioned the Clavien–Dindo classification. We suspect that many of these studies have not used the classification scheme because grade 1 requires no treatment. In a prospective study, Jeong et al. [21] defined complications as abnormal findings of radiologic tests that had been performed when a complication was clinically suspected, which should mean complications of greater than grade 2 by the Clavien–Dindo classification. Our study assessed the severity of postoperative complications after LG, with the rate of severe complications of grade ≥3a being 6.1 % and that of grade ≥3b being 1.3 %. Severe complications can easily lead to surgical mortality. In previous reports, the occurrence of postoperative complications was not found to be associated with the resected area of the stomach [2, 6, 19]. Therefore, on the basis of the complication rate with our surgical techniques, we expect long-term outcomes in the study patients to be favorable.

The prognosis of patients with early gastric cancer is excellent, with a greater than 90 % 5-year survival rate [2]. Multivariate analysis has shown lymph node metastasis to be a significant predictive factor for recurrence [22–24]. In contrast, difficulties and limitations of the technical laparoscopic procedure were detected in lymph node dissection. According to a retrospective study to clarify the technical feasibility and oncologic outcome, cancer recurrence was found in 2.4–4.3 % of patients after curative surgery [6, 18]. There are too few previous reports that show long-term outcome to make valid comparisons, but the 5-year rates of overall survival and recurrence from two previous reports were 90.1 and 93.4 % and 4.2 and 2.5 %, respectively [6, 18]. The 5-year disease-free survival rate for each clinical stage was 99.8 % for stage IA, 98.7 % for stage IB, and 85.7 % for stage II, similar to those of the present study. As for types of recurrence, peritoneal dissemination was reported in 53.3 % of patients, and an average tumor size of 8.3 ± 5.6 cm was critical for recurrence [6]. In addition, not only tumor size (>5 cm) but also a high incidence of the superficially spreading type of early gastric cancer was reported [23]. Indeed, in the present study as well, tumor size in one of the patients with recurrence was 12 cm, and the gastric cancers were the spreading type. Peritoneal dissemination might be related to manipulation by intraoperative forceps, but such manipulation is done not only in the laparoscopic procedures but also in the open surgery procedures [25]. From past studies [24, 26], the number of resected lymph nodes by laparoscopic procedure was >30, and technically this is an adequate number for preventive surgery [26]. Lymph node recurrence was not detected in the present study. However, large tumor size with deep wall invasion would be expected to include serious lymph node metastases. Taken together with peritoneal dissemination, tumor size might be important to determine the indications for LG.

## Conclusion

The present study showed LG to have a safe postoperative course and to benefit oncologic outcomes [27]. In the near future we expect results from a study to expand the indication for LG to advanced-stage cancer to demonstrate efficacy [28].
